# Influence of COVID-19 pandemic and vaccination on the menstrual cycle: A retrospective study in Hungary

**DOI:** 10.3389/fendo.2022.974788

**Published:** 2022-10-27

**Authors:** Klaudia Barabás, Bernadett Makkai, Nelli Farkas, Hanga Réka Horváth, Zsuzsanna Nagy, Kata Váradi, Dóra Zelena

**Affiliations:** ^1^ Institute of Physiology, Medical School, University of Pécs, Pécs, Hungary; ^2^ Centre for Neuroscience, Szentágothai Research Centre, Pécs, Hungary; ^3^ Institute of Bioanalysis, Medical School, University of Pécs, Pécs, Hungary

**Keywords:** COVID-19 vaccines, SARS-Cov-2 infection, COVID-19 pandemic, menstrual cycle, depression, human surveys

## Abstract

Observations of women and clinicians indicated that the prevalence of menstrual cycle problems has escalated during the COVID-19 pandemic. However, it was not clear whether the observed menstrual cycle changes were related to vaccination, the disease itself or the COVID-19 pandemic-induced psychological alterations. To systematically analyze this question, we conducted a human online survey in women aged between 18 and 65 in Hungary. The menstrual cycle of 1563 individuals were analyzed in our study in relation to the COVID-19 vaccination, the COVID-19 infection, the pandemic itself and the mental health. We found no association between the COVID-19 vaccination, the vaccine types or the COVID-19 infection and the menstrual cycle changes. We also evaluated the menstrual cycle alterations focusing on three parameters of the menstrual cycle including the cycle length, the menses length and the cycle regularity in three pandemic phases: the pre-peak, the peak and the post-peak period in Hungary. Our finding was that the length of the menstrual cycle did not change in any of the periods. However, the menses length increased, while the regularity of the menstrual cycle decreased significantly during the peak of the COVID-19 pandemic when comparing to the pre- and post-peak periods. In addition, we exhibited that the length and the regularity of the menstrual cycle both correlated with the severity of depression during the post-peak period, therefore we concluded that the reported menstrual cycle abnormalities during the peak of COVID-19 in Hungary might be the result of elevated depressive symptoms.

## 1 Introduction

Observations of women and clinicians implied that the incidence of menstrual cycle problems has increased during the COVID-19 pandemic. Concerns have been raised in the social media that COVID-19 vaccination may affect the menstrual cycle thereby causing infertility, which increased vaccine hesitancy. Many vaccine sceptics are reluctant to be vaccinated due to the fear from possible side effects of COVID-19 vaccines, hence it is crucial to understand the effects of vaccines – among others – on reproductive health. Since the mRNA vaccine technology is a revolutionary innovation, the least data was accumulated regarding its side-effects (1). Therefore, the new-generation mRNA vaccines were of particular interest in our study. A few reports have already been published investigating the impact of the COVID-19 vaccines on the menstrual cycle (2–4), but systematic analysis was missing at the beginning of our study.

In addition to the COVID-19 vaccines, SARS-CoV-2 infection has also been reported to cause menstrual cycle changes (5).

On the other hand, the COVID-19 crisis has exceedingly increased emotional distress, anxiety, and depression (6–9). It is well known that cortisol, the main stress hormone, inhibits the secretion of gonadotropin releasing hormone that governs the menstrual cycle by its pulsatile release (10). Therefore, the psychological stress experienced during the pandemic such as grief, fear of the virus, social isolation etc., might have contributed to menstrual cycle irregularities. All in all, it was not clear which factor - if any - might be responsible for the menstrual cycle changes.

To examine this question, we conducted an online survey in Hungary to collect information about the menstrual cycle pattern, the received vaccinations, the recognized infection, and the psychological burden of women aged between 18 and 65 during the pandemic. Our study might provide further evidence on the reproductive health safety of COVID-19 vaccines and might help to build trust in vaccines.

## 2 Materials and methods

### 2.1 Participants and study design

We conducted a retrospective analysis focusing on individual’s mental state and menstrual cycle data using quantitative empirical methodology during three stages of the pandemic. We constructed a survey for women which was distributed online in social media using a google form. Thus, our participants were from the entire territory of Hungary. The questionnaire was generated in Hungarian and translated to English so that foreigners living in Hungary could also fill it in. We included the data of foreigners in our study as well because it did not change the outcome of our study.

Women aged between 18 and 65 were recruited between 1 September 2021 and 31 December 2021 to fill out the questionnaire. We collected information of 2429 individuals regarding 3 periods: January 2019 - September 2020 (1); October 2019 – March 2021 (2) and April 2021 – December 2021 (3). The first interval (referred to later as pre-peak) included a pre-pandemic period, the first wave of COVID-19 pandemic in Hungary (04.03.2020 – 17.07.2020.) and a temporary relief period in the summer of 2020. The epidemic curve of the first wave in Hungary was flat with low detected cases reaching the plateau on 4 May with 2055 cases and was mainly localized to hospitals and retirement homes in the capital, Budapest. The second interval (referred to later as peak) examined in our survey was basically the time of the second wave in Hungary (18.07.2020 – 16.02.2021.). The detected cases started to increase in September 2020 with a plateau of 198 785 active cases in December. This was also the time when the Hungarian government applied increasingly strict restrictive measures. The third probed interval (relief, referred to later as post-peak period) in our study coincided with the third wave in Hungary (17.02.2021- 11.06.2021.) that was due to the spreading of the “British variant”. In March 2021 further restrictions were introduced by the government to reduce the risk of catching and spreading of COVID-19. Kindergartens, primary schools, and stores not selling fundamental items were closed for more than a month. However, from 6 April 2021 everyday life started to return to normal as coronavirus-related restrictions were gradually eased when the number of vaccinated Hungarians reached 2.5 million (11).

The questionnaire (available in the **Supplementary Materials**) consisted of questions divided into 84 (C1-84) categories and 6 main categories (I-VI). Some categories were further divided into subcategories (a-c). The first two questions were related to the information sheet. The first category (18 questions) covered demographics and other general data: age, body height, body weight, education, place of residence, employment status, financial situation, coffee, and alcohol consumption, smoking and physical activity. The second section (26 questions) collected information on mental health. The third category contained questions (9) about medication and chronic diseases: thyroid dysfunction, diabetes, high prolactin levels, high blood pressure. The fourth category dealt with female hormone-related questions (12): the time of first menstruation and/or menopause, the number of births, breastfeeding, contraception, menstrual cycle length (i.e., same number of days between the first day of bleeding across consecutive periods) and regularity, menses length (number of days with bleeding within a period), measured hormone levels due to menstrual cycle disturbances (if applicable). The fifth section was about pandemic-related questions (9): previous COVID-19 infection and its severity, vaccination (number of vaccinations, vaccine types) and menstrual abnormalities after vaccination. The last question allowed participants to comment on any of the topics we did not ask for. (The analysis of sociodemographic data is not discussed in this paper.) The ethical approval of the questionnaire has been accepted by the Ethics Committee in Hungary (Medical Research Council; IV/7146- 1 /2021/EKU).

### 2.2 Measures

#### 2.2.1 Mental health test

The overall mental health of the participants was evaluated by a mental health test (MHT) (13), which is based on a short questionnaire. MHT measures global well-being, which is associated with emotional, psychological, social, and spiritual well-being, resilience, coping and savoring capacity, as well as competencies and personality factors that ensure the sustainability of mental health, continuous improvement, and flexibility to adapt to changing conditions. Therefore, a comprehensive picture of the subject’s mental health was determined by measuring five parameters: well-being, savoring, creative-executive efficiency, self-regulation, and resilience. The questionnaire included 17 questions. Responses were given on a 6-point Likert-type scale. The endpoints of the response options were 1 = not at all typical and 6 = very typical.

#### 2.2.2 Shortened Beck Depression Inventory

The presence and the severity of depression symptoms were assessed by a Hungarian version of the shortened Beck Depression Inventory (BDI). This short form of BDI is a 9-item, self-rated scale that measures characteristic symptoms of depression (14, 15). It evaluates social withdrawal, indecisiveness, insomnia, fatigability, somatic preoccupation, work difficulty, pessimism, self-dissatisfaction, and self-accusation. In case of each item a sentence was stated that presented the most severe response such as “I have lost all interest in others.” The participants chose the answer on a 4-point scale ranging from 1 to 4 (not at all typical to very typical) that best described their behavior the month before completing the test. A total score of 0-9 was interpreted “normal”, 10-18 as mild mood disturbance, 19-25 as moderate depression and 26-36 as severe depression.

### 2.3 Statistical analysis

The categorical variables of the questionnaire were characterized with percentage distribution. To determine the connection between the variables Chi-squared test was applied. The variable BDI was treated as a continuous and its relationship with the MHT variables was analyzed using Spearman rank correlation. Significance level was set at 0.05. All calculation were made with SPSS statistical software (IBM Corp. Released 2020. IBM SPSS Statistics for Windows, Version 27.0. Armonk, NY: IBM Corp).

## 3 Results

### 3.1 Anthropometric and other general parameters

**Figure 1:**
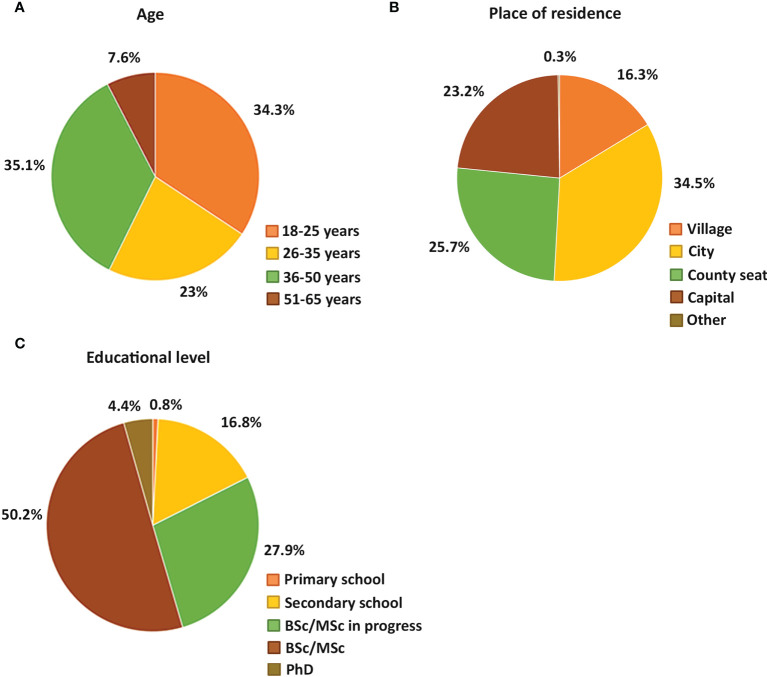
Pie charts show the percentage of different age groups **(A)**, different places of residence **(B)** and different educational background **(C)** of the female participants.

The participants were informed that the survey was anonymous and completely voluntary. 97.6 % of the respondents were Hungarian citizens and 2.4 % were foreigners living in Hungary. After excluding breastfeeding mothers and women using hormonal contraceptive methods, the menstrual cycle changes of 1563 individuals were analyzed. We have categorized the participants into age groups. Based on the database of the Hungarian Central Statistical Office (HCSO) the reproductive age of women is between 15-49 years in Hungary. The lower limit of the first age group was set to 18 years in our study because women between the age of 15 and 18 considered minors in Hungary. Women of reproductive age were further divided into three groups based on the consensus that under 25 years of age, an individual can be considered as young adult, while women between 36-50 years are older adults (16). In addition, one more group was included in the study: women over the reproductive age represented by women between age of 51 and 65. All age groups were represented in the study: 34.3 % of participants was between the age of 18-25, 23 % between the age of 26-35, 35.1 % between 36-50 and 7.6 % between the age of 51-65 (**Figure 1A**). Only the age group of 51-65 was under-represented in the investigation compared to the other age groups in the study, but as members of this group are usually already in menopause, their data was not the most relevant for studying the menstrual cycle parameters by any means. The individuals living in cities, villages, county seats and the capital were also equally represented (**Figure 1B**). However, 82.5 % of the participants was college educated (BSc/MSc or PhD) or undergraduate students (BSc/MSc in progress) meaning that the number of highly qualified individuals were over-represented in the survey (**Figure 1C**).

### 3.2 Association between menstrual cycle changes and COVID-19 vaccination

**Figure 2:**
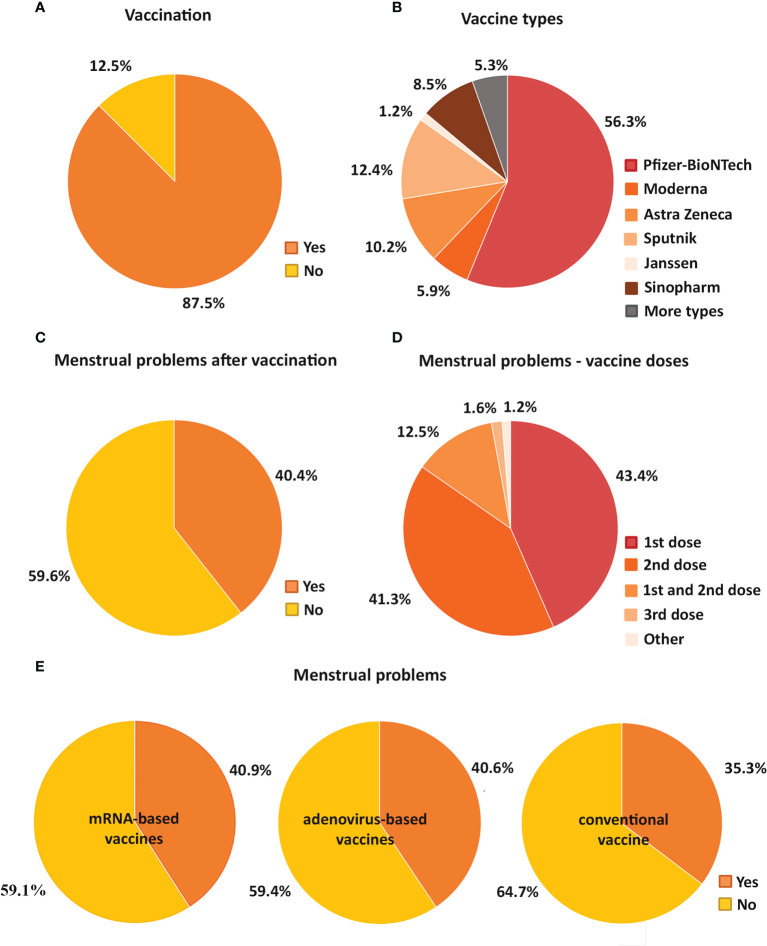
Pie charts demonstrate the percentage of vaccinated and unvaccinated individuals **(A)** and the proportion of the types of vaccines received in the vaccinated group **(B)**. The percentage of participants with and without menstrual cycle problems after vaccination is also shown **(C)**. It is also indicated how many doses of vaccine were given to those with menstrual cycle problems **(D)**. In addition, pie charts show how the proportion of individuals with menstrual problems and without menstrual problems varied for mRNA-based (Pfizer-BioNTech, Moderna), adenovirus-based (AstraZeneca, Sputnik, Janssen) and conventional vaccines (Sinopharm) **(E)**.

The majority of the participants, 87.5 % received at least one vaccination at the time of the examination, while 12.5 % did not receive any vaccination (**Figure 2A**). 62.2 % of the vaccine recipients received mRNA-based vaccine (56.3 % Pfizer-BioNTech, 5.9 % Moderna), 23.8 % received adenovirus vaccine (10.2 % Astra Zeneca, 12.4 % Sputnik, 1.2 % Janssen) and 8.5 % received the traditional, inactivated virus vaccine (Sinopharm), while 5.3 % received more than one type of vaccines (**Figure 2B**). Of the 87.5 % vaccinated individuals, 6.04 % was vaccinated once, 78.04 % was twice and 15.92 % was vaccinated three times.

Regarding the menstrual cycle changes, the menstrual cycle length, the menses length and the regularity of the menstrual cycle were taken into account. 40.4 % of vaccine recipients reported menstrual cycle disturbances after receiving COVID-19 vaccines (**Figure 2C**). Menstrual cycle disturbances were observed after the first and second vaccinations as well. 43.4 % of the participants receiving vaccine experienced menstrual problems after the first, while 41.3 % after the second shot of vaccine. 12.5 % reported menstrual cycle changes after both doses, 1.6 % encountered menstrual problems after the third vaccination only, and 1.2 % after different combinations of the vaccinations (after the first and third, after the second and third and after all vaccine shots) (**Figure 2D**).

Those who had menstrual problems post-vaccination, experienced various problems: both menstrual cycle length shortening (29.9 %) and prolongation (more than 7 days; 22.2 %) was reported. In addition, 13.9 % of female individuals had a missed period post-vaccination, while 7.8 % suffered from prolonged bleeding lasting for more than 2 weeks. The rest of the individuals (26.2 %) had other menstrual problems. The most frequently reported problems included the followings: irregular bleeding (12.2 %), heavier bleeding (4.3 %), strong menstrual cramps (2.8 %) and period reappearance (2 %).

Although a substantial number of vaccine recipients reported that they had experienced menstrual cycle disturbances after receiving vaccines, there was no association found between the vaccination and the menstrual cycle changes (pre-peak period: p=0.81; peak period: p=0.68; post-peak period: p=0.63) (data not shown).

Questions regarding menstruation have been ignored in most large-scale COVID-19 studies (including vaccine trials) (17), which was most critical in case of the newly introduced mRNA-based vaccines. Therefore, we also tested whether the type of vaccines (mRNA-based vaccine, adenovirus vaccine or the inactivated virus vaccine) influenced the occuring cycle changes differently but we did not find any change (pre-peak period: p=0.11; peak period: p=0.13; post-peak period: p=0.24) (**Figure 2E**).

### 3.3 Association between menstrual cycle changes and SARS-CoV-2 infection: *Cross-sectional comparison*

**Figure 3:**
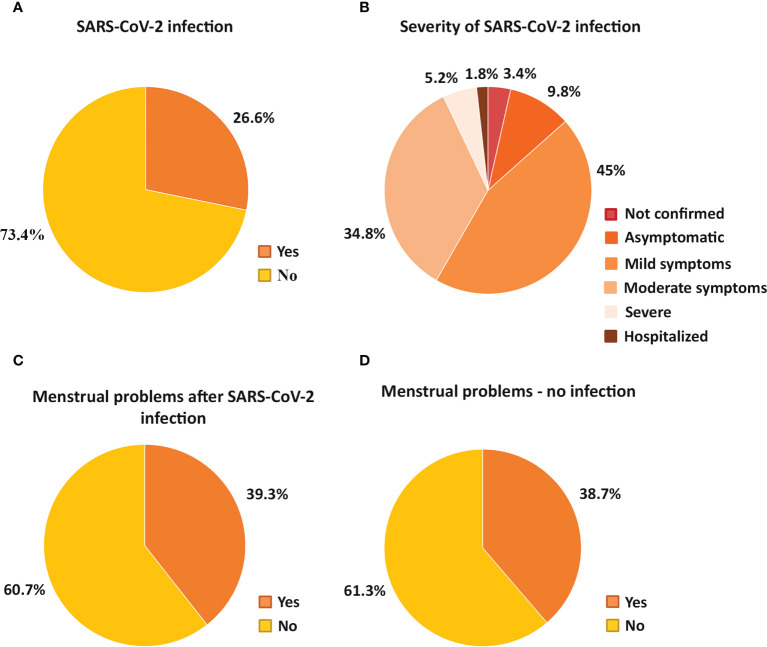
Pie charts show the percentage of participants who were or were not infected with SARS-CoV-2 **(A)**, detailing the proportion of infected individuals based on the severity of the infection **(B)**. The percentage of participants with and without menstrual cycle problems after SARS-CoV-2 infection **(C)** or no infection **(D)** is also demonstrated.

Of those who surveyed, 73.4 % was unaware that they had SARS-CoV-2 infection, while 23.2 % was confirmed to have the infection (**Figure 3A**). Of those who reported to have SARS-CoV-2 infection, 3.4 % did not do a SARS-CoV-2 test but assumed they had the infection, 9.8 % were asymptomatic, 45 % had mild symptoms, 34.8 % had a moderate illness lasting for 7-14 days, 5.2 % had a severe illness lasting for more than two weeks and 1.8 % needed hospitalisation (**Figure 3B**).

We also compared the menstrual cycle changes of the group of women who had SARS-CoV-2 infection with the group who did not get the infection but we found no association between the measured parameters of the menstrual cycle and the SARS-CoV-2 infection (pre-peak period: p=0.37; peak period: p=0.55; post-peak period: p=0.89): the same proportion of individuals reported menstrual cycle problems regardless of SARS-CoV-2 infection (39.3 % of SARS-CoV infected and 38.7 % of uninfected respondents) (**Figures 3C, D**). We also analyzed whether the severity of the SARS-CoV-2 infection was in connection with the menstrual cycle changes but no interaction was uncovered (pre-peak period: p=0.65; peak period: p=0.58; post-peak period: p=0.11) (**Table 1**).

**Table 1:** Correlation between menstrual problems and SARS-CoV-2 infection.

SARS-CoV infection	Participants
Possibly	
Menstrual problems	1.40%
No menstrual problems	2.10%
Asymptomatic	
Menstrual problems	3.50%
No menstrual problems	6.30%
Mild symptoms	
Menstrual problems	16.30%
No menstrual problems	28.50%
Moderate symptoms	
Menstrual problems	13.80%
No menstrual problems	20.60%
Severe symptoms	
Menstrual problems	3.00%
No menstrual problems	2.10%
Hospitalized	
Menstrual problems	1.20%
No menstrual problems	0.70%

Table 1 summarizes the percentage of female participants with and without menstrual problems with SARS-CoV-2 infection of varying severity.

### 3.4 The effect of COVID-19 pandemic on the menstrual cycle: *Longitudinal comparison*

The menstrual cycle problems had also been assessed in the Hungarian population comparing 3 periods: in the pre-peak: January 2019 - September 2020; in the peak: October 2019 – March 2021 and in the post-peak period: April 2021 – December 2021.

**Figure 4:**
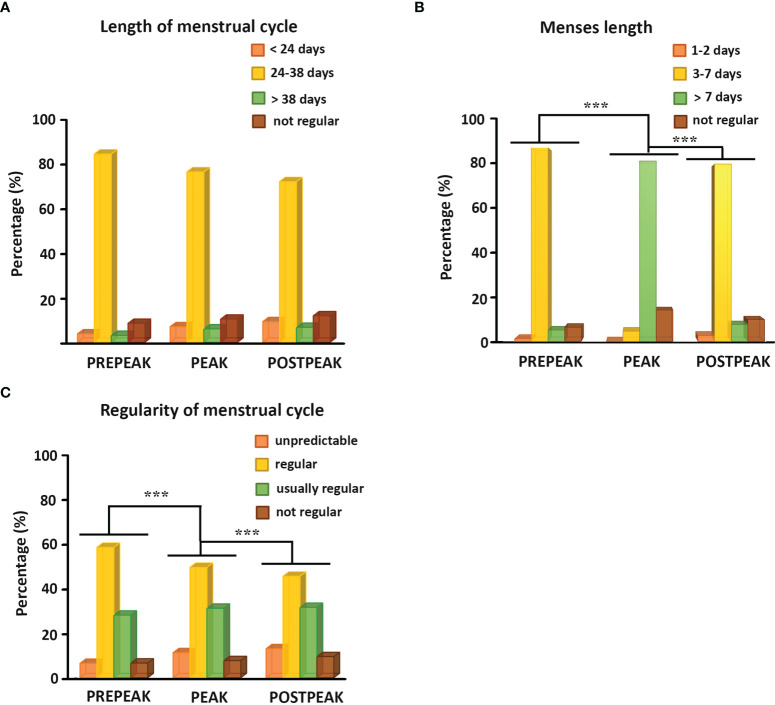
**(A)** Histograms show the percentage of female individuals bleeding more frequently than 24 days, between 24 and 38 days, less frequently than 38 days and having no regular menstrual bleeding during the pre-peak, the peak, and the post-peak period. **(B)** Histograms illustrate the percentage of female participants with menses length lasting for 1-2 days, 3-7 days, more than 7 days or having no regular bleeding during the pre-peak, the peak, and the post-peak period. **(C)** Histograms demonstrate the percentage of female respondents with unpredictable cycle, having regular cycle with a maximum delay of 1-2 days, usually regular cycle with 1-2 missed cycles or 1-2 weeks difference a year, or not regular menstrual cycle during the pre-peak, the peak, and the post-peak period. ***p≤0.001.

We found that the length of the menstrual cycle did not change in any of the study-periods (**Figure 4A**). The vast majority (73-85 %) of female participants had an average cycle length of 24-38 days in all three periods, while shorter or longer menstrual cycles were less common. Interestingly, the menses length increased, while the regularity of the menstrual cycle decreased significantly during the peak of the pandemic compared to the pre- and postpeak periods (p<0.001) (**Figures 4B, C**). The average menses length was more than 7 days long in only 5.1 % of women during the pre-peak, while it lasted longer than 7 days in 81.4 % of women during the peak, then it was restored and only 7.5 % of women had longer period than 7 days during the post-peak period (**Figure 4B**). Although most women had regular periods in all three time periods, their menstrual cycle became more irregular during the peak compared to the pre-peak period and the irregularity further increased thereafter. The start of the period was unpredictable in 6.8 % of the individuals in the pre-peak period, which increased to 11.4 % at the peak and to 13.2 % thereafter. 58.6 % of the participants reported that their menstrual cycles were usually on time, with a maximum delay of 1-2 days (regular cycle) during the pre-peak, while 49.6 % addressed this during the peak and only 45.6 % during the post-peak period. 27.9 % of the respondents answered that they missed only one cycle 1-2 times a year, or there were 1–2-week delays 1-2 times per year in the pre-peak period (usually regular cycle). This proportion increased to 31.3 % at the peak of the pandemic and remained this high (31.7 %) after that as well. During the pre-peak 6.7 % of the participants reported that they had no regular cycle, which increased during the peak and the post-peak period to 7.9 % and 9.5 %, respectively (**Figure 4C**).

### 3.5 Association between menstrual cycle changes and depression

As the COVID-19 pandemic triggered the prevalence of depression and depressive symptoms (6) and it is well-known that depression inhibits the reproductive axis causing menstrual cycle alterations (10), we evaluated whether depressive symptoms developed during the pandemic could be responsible for the high number of noted menstrual cycle problems.

**Figure 5:**
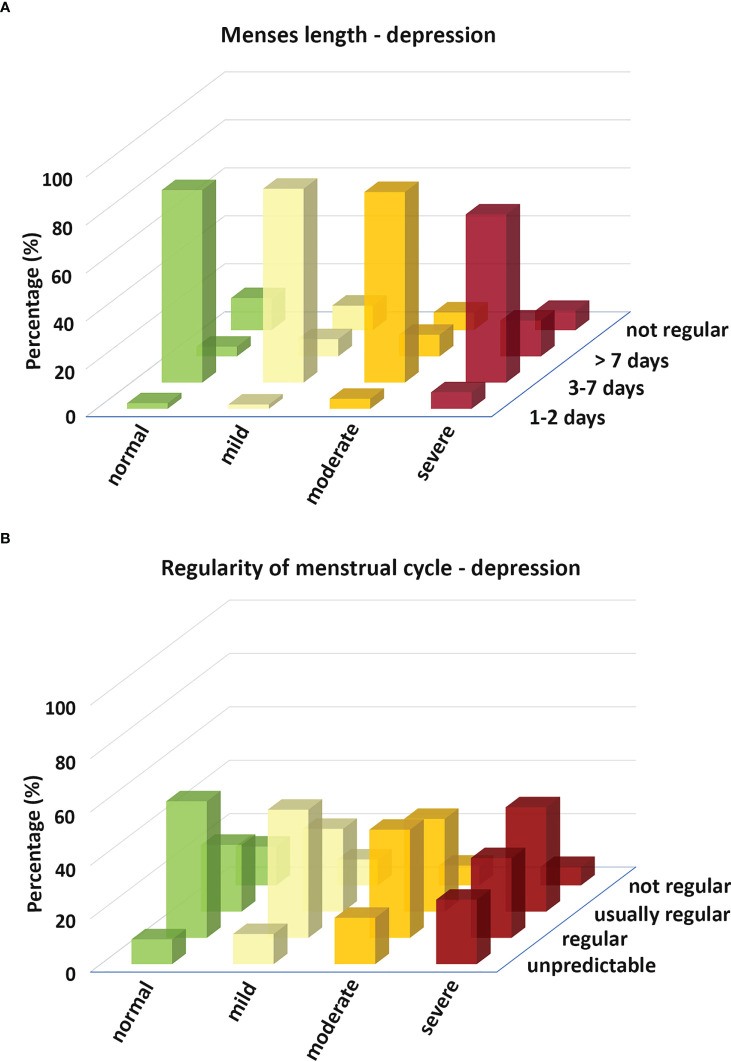
Histograms depict the menses length **(A)** and the regularity of the menstrual cycle **(B)** in relation to the severity of depression determined by the BDI.

Data analysis with Chi-squared test showed that the menses length and the regularity of the menstrual cycle during the post-peak period changed with the severity of depression determined by the BDI. The average menses length (3-7 days) decreased with the severity of depression, while both shortened (1-2 days) and prolonged menses length (more than 7 days) became more frequent (**Figure 5A**). The regularity of the menstrual cycle decreased with the severity of depression (**Figure 5B**).

Although the measured parameters of the MHT (well-being, savoring, creative-executive efficiency, self-regulation, and resilience) did not display association with the surveyed parameters of the menstrual cycle in any of the observed periods, the five measured criteria of the MHT correlated with the result of the BDI. All parameters of the MHT showed negative correlation with the BDI. It means that the more severe the depression is based on the BDI, the lower the values are for the parameters of the MHT. (**Tables 2A**, **B**). We used Spearman’s correlation for the analysis in this case, too.

**Table 2A:** Cross-tabulation analysis between the menses length and BDI.

LengthDepression	1-2 days	3-7 days	<7 days	Not regular	Participants
**Normal**	2.33%	80.23%	4.07%	13.37%	100% (172)
**Mild**	1.79%	80.80%	7.17%	10.24%	100% (948)
**Moderate**	4.18%	79.42%	9%	7.40%	100% (311)
**Severe**	6.90%	70.11%	14.94%	8.05%	100% (87)
**Chi-Square Tests**					
**Pearson Chi-Square**	**Value**	**df**	**Asymptotic Significance (2-sided)**		
	27.023	9	0.001 ***		
**Symmetric Measures**					
**Cramer's V**	**Value**	**Approximate Significance**			
	0.077	0.001

**Table 2B:** Cross-tabulation analysis between the regularity of the menstrual cycle and BDI.

Regularity Depression	Unpredictable	Regular	Usually regular	Not regular	Participants
**Normal**	9.3%	51.16%	25%	14.54%	100% (172)
**Mild**	11.29%	48%	31.01%	9.70%	100% (948)
**Moderate**	17.36%	40.51%	34.73%	7.40%	100% (311)
**Severe**	24.14%	29.89%	39.08%	6.89%	100% (87)
**Chi-Square Tests**					
**Pearson Chi-Square**	**Value**	**df**	**Asymptotic Significance (2-sided)**		
	37.118	9	<0.001 ***		
**Symmetric Measures**					
**Cramer's V**	**Value**	**Approximate Significance**			
	0.09	<0.001			

Table 2 shows the percentage distribution of women with different levels of depression in terms of menses length **(A)** or the regularity of the menstrual cycle **(B)**. The figures in the table show the percentage (and the number) of participants with different menses length and regularity of menstrual cycle. df=degree of freedom, ***p≤0.001.

## 4 Discussion

During the COVID-19 pandemic the prevalence of menstrual cycle problems has increased. The reason for this, however, is still not entirely revealed. The COVID-19 vaccines caused considerable concern because of a potential disruption of the menstrual cycle. In addition, growing evidence suggests that SARS-CoV-2 infection may have an impact on the menstrual cycle (5, 18). It is also clear that the COVID-19 pandemic put a great psychological burden on the society increasing the level of depression that can also influence the menstrual cycle (19). Thus, our survey aimed to explore whether the menstrual cycles of women between the age of 18-65 have been affected by the COVID-19 vaccines, SARS-CoV-2 infection, the COVID-19 pandemic, or psychological distress.

Human studies so far have shown that COVID-19 vaccines have a subtle and reversible effect on the menstrual cycle. Menstrual abnormalities such as menstrual irregularities, increase in the cycle length, menses length and heavier menstruation were observed post-vaccination. However, these changes were no greater than normal fluctuations and were restored within a few months (2–4, 20). Animal experiments also confirmed the lack of substantial vaccine effect on reproduction. No effects were found on fertility, or any studied ovarian and uterine parameters (12).

The occurrence of menstrual disturbances after COVID-19 vaccinations is not that surprising as vaccination has been linked to menstrual cycle changes earlier. It was published that the human papilloma virus vaccine had caused irregular and abnormal amount of menstrual bleeding (21). Such menstrual abnormalities can be the result of inflammatory reactions (22). Because of the severe symptoms and the rapid spread of SARS-CoV-2, the COVID-19 vaccines were developed and approved hastily. In addition, a new class of vaccines, the mRNA-based vaccines were also introduced against the SARS-CoV-2. The rapid development of vaccines has not allowed extensive studies of all the side effects, especially for mRNA-based vaccines, which were not yet used before. Therefore, it was not assessed whether immunological reactions associated with vaccines may affect women’s reproductive health. Lipid nanoparticles (LNPs) for instance that protect mRNA from degradation and help to deliver mRNA into the cells have been reported to be highly inflammatory in mice (23). Since the menstruation itself is associated with increased inflammation, we hypothesized that the LNPs being inflammatory and lipophilic molecules may interact with lipophilic sexual hormones and affect the menstrual cycle.

Our study demonstrated that 40.4 % of vaccine recipients had complained of menstrual cycle problems, particularly after the first and the second dose of COVID-19 vaccines. Despite of the fact that a large number of individuals reported menstrual cycle abnormalities, we found no correlation between the impact of vaccination and the menstrual cycle disturbances. According to our assumption we also tested whether the type of COVID-19 vaccines, especially the mRNA-based vaccines affected the menstrual cycle differently but we found no proof of that. However, we should mention that the slight and temporary menstrual cycle changes observed in previous studies (2–4, 20) could be masked by the over-representation of the highly qualified individuals (82.4 %) in our sample since the prevalence of irregular menstruation is increased in women with low educational levels (24). Also, menstrual cycle length variations occur more frequently with increasing age in women from lower social groups (25), whose representation was insufficient in our study.

SARS-CoV-2 infection may have also accounted for the observed menstrual cycle abnormalities during the pandemic (17). There is still relatively little scientific data available on how and to what extent COVID-19 infection may affect the menstrual cycle. It has been published that women with severe COVID-19 symptoms are more likely to have menstrual cycle problems (5). Additionally, a case study reported that a 27-year-old female patient developed amenorrhea during and after a mild form of SARS-CoV-2 infection (26). SARS-CoV-2 infection could influence the menstrual cycle by influencing the hypothalamic-pituitary-gonad axis (17), but could also have a more specific effect on the reproductive system. The SARS-CoV-2 can bind to the angiotensin-converting enzyme ACE2, which acts as a viral receptor and is also expressed in the endometrium (27, 28). As ACE2 has a key role in regulating vasoconstriction of the arteries that induces menstruation, the alteration of ACE2 may cause menstrual cycle abnormalities. Even though available data and scientific facts suggest that SARS-CoV-2 infection may alter the menstrual cycle, we found no connection between the SARS-CoV-2 infection or its severity and menstrual cycle disruptions.

Finally, we examined the effect of the pandemic itself on the menstrual cycle with its possible depression-inducing potential. Interestingly, the peak of the infection was associated with longer menses length and more irregularity in the menstrual cycle, which was normalized after the relief. Menstrual cycle characteristics such as cycle length, regularity, and menses length show strong association with psychiatric disorders including depression (29, 30).

Women during their reproductive years are nearly twice as likely to develop depression as men (31). In depression, the corticotropin-releasing hormone (CRH) levels, and consequently the cortisol levels are elevated resulting in the inhibition of the action of gonadotropin-releasing hormone (GnRH) neurons, gonadotrophs, and gonads (10). The COVID-19 pandemic increased the rate of depression (6) and exacerbated the existing mental health problems (7). Loneliness due to social distancing (8), elevated levels of fear of SARS-CoV-2 infection (9), or loss and grief during the pandemic became major factors contributing to the development of depressive symptoms. As a strong association between depression and menstrual cycle disorders was noted, we examined whether there was a connection between the depressive symptoms and the menstrual cycle disturbances.

We found an association between the BDI scores and the length and the regularity of the menstrual cycle during the post-peak period. The severity of the depression based on BDI positively correlated with the menses length changes (shortening and prolongation) and the irregularity of the menstrual cycle. Although the MHT did not show connection with the menstrual cycle changes, it exhibited negative correlation with the BDI. Individuals who scored lower in the MHT, which represents a general mental health state, had increased values in the BDI, which evaluated their mental health a month before completing the test. Although we could not measure, we assumed that the rate of depression might have been elevated during the peak of the pandemic. This suggests that the menstrual cycle problems observed during the peak of infection, the significant increase in the menses length and the irregularity of the menstrual cycle, may be a consequence of depression.

Our survey has revealed a connection between depressive-like symptoms and menstrual cycle alterations but found no evidence of correlation between post-vaccination or SARS-CoV-2 infection and menstrual cycle changes. It suggests that depression may be a major factor causing menstrual cycle abnormalities during the COVID-19 pandemic.

## 5 Limitations

Our study has pitfalls and limitations. We should be cautious about drawing general conclusions from the gathered data as the questionnaire was completed voluntarily by female individuals online. Therefore, the collected data may contain some bias such as social acceptance error. Recall bias may also be a problem since the study was self-reporting and asked questions for an interval of more than one year. Furthermore, the over-representation of highly qualified individuals may also lead to bias as women’s reproductive health is highly influenced by their socioeconomic status. Lower educational level for instance has been shown to act as a factor promoting irregular menstruation (24). Another drawback of our study is that we could not follow the level of depression of the female participants separately during the pre-peak, the peak, and the post-peak period. We could only assess their general mental health and the rate of depression at the end of the post-peak period. Therefore, we could only make assumptions that the depression level was the greatest during the peak period of the pandemic.

## 6 Conclusions

Our study provides evidence on the reproductive health safety of COVID-19 vaccines and indicates that the effect of COVID-19 vaccines and SARS-CoV-2 infection on the menstrual cycle may be negligible compared to the effect of depression.

## Data availability statement

The original contributions presented in the study are included in the article/**Supplementary Material**. Further inquiries can be directed to the corresponding author.

## Ethics statement

The ethical approval of the questionnaire has been accepted by the Ethics Committee in Hungary (Medical Research Council; IV/7146- 1 /2021/EKU).

## Author contributions

DZ: Conceptualization, methodology, investigation. BM and NF: Statistical analysis. ZN: Conceptualization, clinical expertise. KB: Investigation, data assessment, writing. HH and KV: Visualization. All authors contributed to the article and approved the submitted version.

## Funding

Project no. TKP2021-EGA-16 has been implemented with the support provided from the National Research, Development and Innovation Fund of Hungary, financed under the TKP2021-EGA funding scheme. This study was also supported by the National Research Development and Innovation Office of Hungary (grant numbers K141934, K138763, and K120311).

## Conflict of interest

The authors declare that the research was conducted in the absence of any commercial or financial relationships that could be construed as a potential conflict of interest.

## Publisher’s note

All claims expressed in this article are solely those of the authors and do not necessarily represent those of their affiliated organizations, or those of the publisher, the editors and the reviewers. Any product that may be evaluated in this article, or claim that may be made by its manufacturer, is not guaranteed or endorsed by the publisher.
