# Supplementary material for: Influence of COVID-19 pandemic and vaccination on the menstrual cycle: A retrospective study in Hungary
Source: Front Endocrinol (Lausanne). 2022 Oct 27;13:974788. doi: 10.3389/fendo.2022.974788 (PMC9646704; doi:10.3389/fendo.2022.974788)
Supplement: Supplementary file 1 [file Table_1.docx]

**Supplementary material**

**Questionnaire**

**The impact of mental health on the menstrual cycle during the pandemic**

We invite you to participate in our research by filling out this questionnaire. Participation in the research is entirely voluntary. Before you decide, we would like to share some research-related information with you about why we find this research and your participation important, and what your involvement means. Please take the time to read the information sheet and contact us if you have any questions or if anything is unclear. It takes about five minutes to read the information sheet.

**C1**. 1. Do you want to view the detailed research information?

-Yes

-No

INFORMATION SHEET

QUESTIONNAIRE FOR PARTICIPANTS

Version 1.0, dated 18/08/2021

Title of the research: The impact of mental health on the menstrual cycle during the pandemic

Name and contact details of the research leader:

Dr. Dóra Zelena

University of Pécs, Medical School, Institute of Physiology

dora.zelena@aok.pte.hu

+ 36-72-536-243

Research ethics authorization number: IV / 7146-1 / 2021 / EKU

We invite you to participate in our research by filling out this questionnaire. Participation in the research is entirely voluntary. Before you decide, we would like to share some research-related information with you about why we find this research and your participation important, and what your involvement means. Please take the time to read the information sheet and contact us if you have any questions or if anything is unclear. It takes about five minutes to read the information sheet.

What is the goal of the research?

The aim of our research is to assess the mental stress caused by the pandemic and how it affected the course of the menstrual cycle.

In the present study, we address women between the ages of 18 and 65.

What do you have to know about participating in this study?

If you choose to participate, please complete an online questionnaire. This takes about 10 minutes. The questionnaire asks questions about some of your habits, in addition to general demographics, and includes a previously validated 18-question MHT (Mental Health Test) and a 9-question Beck Depression test to measure mental health. You will also need to answer questions about your period and how the epidemic affected you. Most questions are single choice questions, but there are also some free-text questions that allow you to refine your answers if needed.

Costs and payments

Participation in the research is free. We cannot offer payment for participation.

What data will be collected and / or analyzed?

We only collect data using the online questionnaire. Only pooled data that does not identify you and your responses will be analyzed and published.

What are the possible disadvantages, burdens, and risks of participation (if there is any)?

Apart from the time required to complete the questionnaire, we do not anticipate any other burden or risk arising from participation.

What are the potential benefits of participation (if there is any)?

There is no direct personal benefit of participating in the research, but it contributes to a better understanding of the effects of the pandemic.

Is my data or information given by me kept confidential?

The questionnaire is filled in anonymously, the answers cannot be linked to you in any way.

Can I terminate my participation in the research?

You can stop completing the questionnaire at any time before submitting your information. Once the data has been sent, it is not possible to change it.

Who approved the survey?

The research was reviewed and approved by the Scientific and Research Ethics Committee of the Health Science Council.

Acknowledgement

Thank you for taking the time to read this information material. We hope you decide to participate in the research!

Other things to do

If you agree to participate in this study, we will ask you to complete the online statement of consent dated 18/08/2021, version 1.0, before completing the questionnaire.

2. GDPR

• I have read and understood the information sheet dated 18/08/2021 (version 1.0) or the project was fully explained to me by the researchers.

• I was given the opportunity to ask questions about the project and received satisfactory answers.

• I am aware that my participation is voluntary. I also understand that I may suspend / withdraw my participation at any time until I submit my answers. I do not have to explain why I do not want to participate further and there will be no adverse consequences if I choose to stop completing the questionnaire.

• I understand that the data collected during the research will be processed in accordance with data protection laws, as described in the Information Sheet.

**C2.** I agree to participate in the above research.

-Yes

-No

For completing the questionnaire, "pandemic" means the period from September 2020 to March 2021. "Before the pandemic" covers the period from January 2019 to September 2020 and "After the pandemic" covers the period after March 2021.

1. **Demographics and other general data**

**C3.** 3. Your age:

-18-25 years

-26-35 years

-36-50 years

-51-65 years

**C4.** 4. Your body height: ___ cm

**C5.** 5. Your body weight

**C5a.** a. before the pandemic (January 2019-September 2020): _______ kg

**C5b.** b. currently: ____________kg

**C6.** 6. Your highest level of education:

basic degree (8 general)

-mediate (graduation)

in progress to get BSc or MSc degree

College (BSc)

University (MSc)

-PhD

**C7.** 7. Your place of residence

a. its location

-village

-city

-county seat

-capital

-other

**C8.** b. Who do you live together in one household?

-alone

-with family

-other (friends, college, etc.)

**C9.** c. Has your place of residence changed since the pandemic?

did not change

You live in another settlement

You live in one household with other people

**C10.** If you live in a different settlement than before the pandemic, then ...

-the current is smaller

-the current is larger

**C11.** If you live in one household with other people than before the pandemic, then ...

- less people live together than before

-more people live together than before

- the same number of people live together than before

**C12.** If you live in one household with other people than before the pandemic, then you live ...

-in a smaller apartment

-in a larger apartment

-in a same-size apartment

**C13.** 8. You are:

-employed

-entrepreneur

-unemployed

-University student

-other, ………………………………..

**C13a.**Your workplace during the pandemic

-has not changed

-got better

-lost your job

**C14.** 9. What is your subjective financial situation?

-below average

-average

-above average

Here you can write any other comments about your general life situation, its change during the pandemic: _______

**C16.** 10. Do you regularly consume coffee?

-no

-0-1 cups / day

-2-3 cups / day

-more than 3 cups a day

**C16a.** Has this changed since the pandemic?

-no

-currently consuming more

-currently consuming less

**C17.** b. Do you regularly consume alcohol?

-yes

-occasionally

-no

**C17a.** Has this changed since the pandemic?

-no

-currently consuming more

-currently consuming less

**C18.** c. Do you smoke?

-yes

-occasionally

-no

**C18a.** Has this changed since the pandemic?

-no

-currently smoking more

-currently smoking less

**C19.** 11. Do you perform physical activity regularly (e.g., walking, training):

-no

-1-3 hours / week

-4-6 hours / week

-more than 6 hours a week

**C19a.** Has this changed since the pandemic?

-no

-currently more active

-currently less active

**C20.** Here you can write any other comments about changes in your habits during the pandemic: _______

1. **Mental health**

For each of the following statements, please indicate how specific it is to you. *

1-Not at all characteristic, 2-Not characteristic, 3-Slightly characteristic, 4-Characteristic, 5-Very characteristic, 6-Completely characteristic

**C21.** 12. In my daily life, there is more joy than sorrow

**C22.** 13. I easily become impatient

**C23.** 14. I can easily bring back the joy of pleasant memories of the past

**C24.** 15. I recover soon after difficult times

**C25.** 16. I often have ideas that others can relate to and make them think further

**C26.** 17. According to others I am a good at solving problems

**C27.** 18. I am hasty-tempered (I act first and think afterwards)

**C28.** 19. I can easily achieve my goals

**C29.** 20. I like to collect good memories and recall them later

**C30.** 21. I recover quite quickly after going through difficult times

**C31.** 22. I can make myself feel positive when I imagine that the future will be happy

**C32.** 23. It usually takes a while for me to move on after experiencing difficult moments

**C33.** 24. My mental health is good

**C34.** 25. I am good at jobs where new and original ideas are needed

**C35.** 26. I will be upset if something does not turn out the way I planned

**C36.** 27. I am good at picking up on how other people are feeling or what they are thinking

**C37.** 28. All in all, how happy would you say you are (1. not at all, 6. very much)?

Please indicate which of the following statements describe your feelings most over the past months!

1-Not typical at all, 2-Hardly typical, 3-Typical, 4-Very typical

**C38.** 29. I lost all interest in others

**C39.** 30. I can't decide anything anymore

**C40.** 31. I wake up several hours earlier than usual and can’t fall asleep again

**C41.** 32. I am too tired to do anything

**C42.** 33. I’m so worried about physical complaints that I can’t think of anything else

**C43.** 34. I can't do any work

**C44.** 35. I see my future hopeless and I feel my situation will not change.

**C45.** 36. I am unsatisfied with everything, or I am indifferent.

**C46.** 37. I constantly blame myself.

1. **Medications and chronic diseases**

**C47.** 38. Have you been diagnosed with any mental health problems?

-yes

-no

If yes, please briefly describe the problem and indicate the approximate date of diagnosis:

**C48.** Other comments about the mental condition: _______

**C49.** Do you have a diagnosed thyroid dysfunction? *

no

yes

**C49a.** If you were diagnosed with thyroid disorder, what was the diagnosis?

Hypothyroidism

Hyperthyroidism

**C49b.** If you were diagnosed with thyroid disorder, is it treated?

Yes, the treatment is well adjusted

needs constant medical supervision

**C49c.** If you were diagnosed with thyroid dysfunction, when was it detected?

it was detected before the pandemic

it was detected during the pandemic (September 2020-March 2021)

it was detected after March 2021

**C50.** Do you have diagnosed diabetes? *

no

yes

**C50a.** If you were diagnosed with diabetes, what type is it?

Type 1

Type 2

**C50b.** If you were diagnosed with diabetes, is it treated?

Yes, the treatment is well adjusted

needs constant medical supervision

**C50c.** If you were diagnosed with diabetes, when was it detected?

it was detected before the pandemic

it was detected during the pandemic (September 2020-March 2021)

it was detected after March 2021

**C51.** Do you have known high prolactin levels? *

no

yes

**C51a.** If you have known high prolactin levels, is it treated?

Yes, the treatment is well adjusted

needs constant medical supervision

**C51b.** If you have known high prolactin levels, when was it detected?

it was detected before the pandemic

it was detected during the pandemic (September 2020-March 2021)

it was detected after March 2021

**C52.** Do you have known high blood pressure?

No

Yes

**C52a.** If you have known high blood pressure, is it treated?

Yes, the treatment is well adjusted

needs constant medical supervision

**C52b.** If you have known high blood pressure, when was it detected?

it was detected before the pandemic

it was detected during the pandemic (September 2020-March 2021)

it was detected after March 2021

**C53.** Do you have any other known diseases?

no

yes

**C53a.** If you have any other known diseases, please specify:

**C53b.** If you have any other known diseases, is it treated?

Yes, the treatment is well adjusted

needs constant medical supervision

**C53c.** If you have any other known diseases, when was it detected?

it was detected before the pandemic

it was detected during the pandemic (September 2020-March 2021)

it was detected after March 2021

**C54.**Do you take any medication regularly?

no

yes

other

**C54a.** Please list your medicines:

**C55.** You can write other comments about your health here:

**IV. Female sex hormones-related questions**

39. How old were you at the time of

**C56.** a. first menstruation: ___ years

**C57.** b. menopause: ____ years, if relevant

**C58.** 40. Number of births:

-0

-1

-2

-more than 2

**C59.** If you have given birth at least once, when was the last time you gave birth?

-Before the pandemic (before September 2020)

-During the pandemic (between September 2020-March 2021)

- After March 2021

**C60.** 41. Did you breastfeed before the pandemic (between January 2019 and March 2020)?

-yes

-no

**C61.** Did you breastfeed during the pandemic (September 2020-March 2021)?

-yes

-no

**C62.** Are you breastfeeding now? *

-yes

-no

**C63.** 42. Have you used any of the following methods of contraception in the last year? *

You can mark more than one answer if you have used more than one method.

-Contraceptive pill

-Contraceptive injection

-Contraceptive implant

-Contraceptive patch

-Vaginal ring

-Hormonal spiral

-I don't use any of them

**C64.** How often did you have your period before the pandemic (January 2019-September 2020)?

More often than 24 days

Between 24-38 days

Less than 38 days

I didn't have regular bleeding

**C65.** How often did you have your period during the pandemic (September 2020-March 2021)?

More often than 24 days

Between 24-38 days

Less than 38 days

I didn't have regular bleeding

**C66.** How often do you have your period since March 2021?

More often than 24 days

Between 24-38 days

Less than 38 days

I didn't have regular bleeding

**C67.** How many days did your period usually last before the pandemic (January 2019-September 2020)?

1-2 days

3-7 days

More than 7 days

I didn't have regular bleeding

**C68.** How many days did your period usually last during the pandemic (September 2020-March 2021)?

1-2 days

3-7 days

More than 7 days

I didn't have regular bleeding

**C69.** How many days did your period usually last since March 2021?

1-2 days

3-7 days

More than 7 days

I didn't have regular bleeding

**C70.** How regular was your menstrual cycle before the pandemic (January 2019-September 2020)?

It was usually on time, with a maximum delay of 1-2 days.

It was usually regular, I missed only 1 cycle 1-2 times a year, or there were 1-2 week delays 1-2 times per year.

It was unpredictable when my period started.

I didn't have regular bleeding

**C70a.** If your period was not on time before the pandemic, did you know the reason?

Yes, there was a delay, when I was under a lot of stress or I intensely worked out

I didn't know the reason.

Other:

**C71.** How regular was your menstrual cycle during the pandemic September 2020-March 2021)?

It was usually on time, with a maximum delay of 1-2 days.

It was usually regular, I missed only 1 cycle 1-2 times a year, or there were 1-2 week delays 1-2 times per year.

It was unpredictable when my period started.

I didn't have regular bleeding

**C71a.** If your period was not on time during the pandemic, did you know the reason?

Yes, there was a delay, when I was under a lot of stress or I intensely worked out

I didn't know the reason.

Other:

**C72.** How regular was your menstrual cycle since March 2021?

It was usually on time, with a maximum delay of 1-2 days.

It was usually regular, I missed only 1 cycle 1-2 times a year, or there were 1-2 week delays 1-2 times per year.

It was unpredictable when my period started.

I didn't have regular bleeding

**C72a.** If your period was not on time, did you know the reason?

Yes, there was a delay, when I was under a lot of stress or I intensely worked out

I didn't know the reason.

Other:

**C73.** My missed menstrual bleeding reappeared:

irrelevant

before the pandemic

during the pandemic

after COVID-19 infection

after the first vaccine

after the second vaccine

after the third vaccine

**C74.** Have you had hormone levels measured because of cycle disorder?

no

yes

**C74a.** If yes, please provide the measured values and the date of the test.

We are interested in the values of the following hormones: LH, FSH, estradiol, prolactin, testosterone, TSH, Cortisol

**C75.** Here you can write other comments about your menstrual cycle and hormonal system:

**V.Pandemic-related questions**

**C76.** Have you had COVID infection?

Not to my knowledge

I went through it asymptomatically

Yes, with a mild, short-term illness

Yes, with moderate illness lasting 7-14 days

Yes, with a serious illness lasting more than two weeks

Yes, I needed hospital care

Yes, I needed a ventilator

Other:

**C77.** Have you been vaccinated against COVID-19?

no

yes

**C78.** If so, which type? *

I did not get

Pfizer-BioNTech

Modern

Astra Zeneca

Sputnik (Russian)

Sinopharm (Chinese)

I got several vaccines

Other:

**C79.** If so, when did you get the first vaccination?

**C80.** If so, when did you get the second vaccination?

**C81.** If so, when did you get the third vaccination?

**C82.** Have you experienced menstrual disorder since the vaccination?

I didn't get vaccinated.

I did not experience menstrual disorder.

Yes, I have experienced menstrual disorder.

**C82a.** If you had experienced a disturbed menstrual cycle, what was the problem?

I missed one period.

My period was more than seven days late.

My period started earlier than before.

My period was prolonged, it lasted longer than 2 weeks.

Other:

**C83.** Other comments about COVID-19 infection and / or vaccination:

**C84.** If you have any comments on this topic that we didn't ask for, please let us know here:
